# Generating relations and other results associated with some families of the extended Hurwitz-Lerch Zeta functions

**DOI:** 10.1186/2193-1801-2-67

**Published:** 2013-02-25

**Authors:** Hari M

**Affiliations:** Department of Mathematics and Statistics, University of Victoria, Victoria, British Columbia V8W 3R4 Canada

**Keywords:** Riemann, Hurwitz (or generalized) and Hurwitz-Lerch Zeta functions, Lerch Zeta function and the Polylogarithmic (or de Jonquière’s) function, General Hurwitz-Lerch Zeta function, Gauss and Kummer hypergeometric functions, Fox-Wright *Ψ*-function and the $\bar{H}$-function; Mittag-Leffler type functions, Mellin-Barnes type integral representations and Meromorphic continuation, Generating functions and Eulerian Gamma-function and Beta-function integral representations

## Abstract

Primary 11M25, 33C60; Secondary 33C05

## Introduction and preliminaries

Throughout our present investigation, we use the following standard notations:

$$N:=\{1,2,3,\cdots \;\},\;\;N_{0}:=\{0,1,2,3,\cdots \;\}=N\cup \{0\}$$

and

$$Z^{-}:=\{-1,-2,-3,\cdots \;\}=Z_{0}^{-}\setminus \{0\}.$$

Here, as usual,Z denotes the set of integers,R denotes the set of real numbers, $R^{+}$ denotes the set of *positive* real numbers andC denotes the set of complex numbers.

The familiar general Hurwitz-Lerch Zeta function Φ(*z*, *s*, *a*) defined by (see, for example, (Erdélyi *et al.*^1953^, p. 27. Eq. 1.11 (1)); see also Srivastava and Choi ((2001, p. 121 *et seq.*) and (Srivastava and Choi 2012), p. 194 *et seq.*)

1.1 $$\begin{array}{l} \;\Phi (z,s,a):=\overset{\infty }{\underset{n=0}{\sum }}\frac{z^{n}}{{\left(n+a\right)}^{s}}\;\\ \;\left(a\in C\setminus Z_{0}^{-};\;s\in C\;\;\text{when}\;\;|z|<1;\;ℜ(s)>1\;\;\text{when}\;\;|z|=1\right)\; \end{array}$$

contains, as its *special* cases, not only the Riemann Zeta function *ζ*(*s*), the Hurwitz (or generalized) Zeta function *ζ*(*s*, *a*) and the Lerch Zeta function *ℓ*_*s*_(*ξ*) defined by (see, for details, (Erdélyi *et al.*^1953^, Chapter I) and Srivastava and Choi ((2001), Chapter 2)

1.2 $$\begin{array}{l} \zeta (s)\;:=\;\overset{\infty }{\underset{n=0}{\sum }}\frac{1}{{\left(n+1\right)}^{s}}=\Phi (1,s,1)=\zeta (s,1)\;\left(ℜ(s)>1\right), \end{array}$$

1.3 $$\begin{array}{cc} \;\zeta (s,a) & :=\overset{\infty }{\underset{n=0}{\sum }}\frac{1}{{\left(n+a\right)}^{s}}=\Phi (1,s,a)\;\left(ℜ(s)>1;\;a\in C\setminus Z_{0}^{-}\right) \end{array}$$

and

1.4 $$\;\begin{array}{c} \ell_{s}(\xi ):=\overset{\infty }{\underset{n=0}{\sum }}\frac{e^{2n\pi i\xi }}{{\left(n+1\right)}^{s}}=\Phi \left(e^{2\pi i\xi },s,1\right)\;\left(ℜ(s)>1;\;\xi \in R\right), \end{array}$$

respectively, but also such other important functions of *Analytic Number Theory* as the Polylogarithmic function (or *de Jonquière’s function*) Li_*s*_(*z*):

1.5 $$\begin{array}{l} {\text{Li}}_{s}(z):=\overset{\infty }{\underset{n=1}{\sum }}\frac{z^{n}}{n^{s}}=\mathrm{z\Phi}(z,s,1)\;\\ \left(s\in C\;\text{when}\;\left|z\right|<1;\;ℜ(s)>1\;\text{when}\;\left|z\right|=1\right)\; \end{array}$$

and the Lipschitz-Lerch Zeta function *ϕ*(*ξ*, *a*, *s*) (see Srivastava and Choi ((2001), p. 122, Equation 2.5 (11))):

1.6 $$\begin{array}{l} \phi (\xi ,s,a):=\overset{\infty }{\underset{n=0}{\sum }}\frac{e^{2n\pi i\xi }}{{\left(n+a\right)}^{s}}=\Phi \left(e^{2\pi i\xi },s,a\right)\;\\ \left(a\in C\setminus Z_{0}^{-};\;ℜ(s)>0\;\;\text{when}\;\;\xi \in R\setminus Z;\;ℜ(s)>1\right.\;\;\;\\ \left.\text{when}\;\;\xi \in Z\right),\; \end{array}$$

which was first studied by Rudolf Lipschitz (1832-1903) and Matyáš Lerch (1860-1922) in connection with Dirichlet’s famous theorem on primes in arithmetic progressions (see also (Srivastava 2011), Section 5). Indeed, just as its aforementioned special cases *ζ*(*s*) and *ζ*(*s*, *a*), the Hurwitz-Lerch Zeta function Φ(*z*, *s*, *a*) defined by (1.1) can be continued *meromorphically* to the whole complex *s*-plane, except for a simple pole at *s* = 1 with its residue 1. It is also known that (Erdélyi *et al.*^1953^, p. 27, Equation 1.11 (3))

1.7 $$\begin{array}{l} \;\Phi (z,s,a)=\frac{1}{\Gamma (s)}\int_{0}^{\infty }\frac{t^{s-1}\;e^{-\text{at}}}{1-ze^{-t}}\;dt\;\\ \;\;\;\;\;=\frac{1}{\Gamma (s)}\int_{0}^{\infty }\frac{t^{s-1}\;e^{-(a-1)t}}{e^{t}-z}\;dt\;\\ \;\left(ℜ(a)>0;\;ℜ(s)>0\;\;\text{when}\;\;|z|≦1\;(z\neq 1);\;ℜ(s)>1\right.\;\;\;\\ \left.\;\text{when}\;\;z=1\right).\; \end{array}$$

Making use of the Pochhammer symbol (or the *shifted* factorial) ${\left(\lambda \right)}_{\nu }\;\;(\lambda ,\nu \in C)$ defined, in terms of the familiar Gamma function, by

1.8 $$\;\begin{array}{ll} \;{\left(\lambda \right)}_{\nu }: & =\frac{\Gamma (\lambda +\nu )}{\Gamma (\lambda )}\\ =\left\{\begin{array}{cc} 1 & \;(\nu =0;\;\lambda \in C\setminus \{0\})\\ \lambda (\lambda +1)\cdots (\lambda +n-1) & \;(\nu =n\in N;\;\lambda \in C), \end{array}\right. \end{array}$$

it being understood *conventionally* that (0)_0_: = 1 and assumed *tacitly* that the Gamma quotient exists, we recall each of the following well-known expansion formulas:

1.9 $$\zeta (s,a-t)=\overset{\infty }{\underset{n=0}{\sum }}\frac{{\left(s\right)}_{n}}{n!}\;\zeta (s+n,a)t^{n}\;(|t|<|a|)$$

and

1.10 $$\Phi (z,s,a-t)=\overset{\infty }{\underset{n=0}{\sum }}\frac{{\left(s\right)}_{n}}{n!}\;\Phi (z,s+n,a)t^{n}\;(|t|<|a|).$$

More generally, it is not difficult to show similarly that

1.11 $$\;\begin{array}{cc} \;\overset{\infty }{\underset{n=0}{\sum }}\frac{{\left(\lambda \right)}_{n}}{n!}\;\Phi (z,s+n,a)t^{n} & =\overset{\infty }{\underset{k=0}{\sum }}\frac{z^{k}}{{\left(k+a\right)}^{s-\lambda }{\left(k+a-t\right)}^{\lambda }}\\ =:\vartheta_{\lambda }(z,t;s,a)\;(|t|<|a|), \end{array}$$

which would reduce immediately to the expansion formula (1.10) in its special case when *λ* = *s*. Moreover, in the limit case when

$$t\mapsto \frac{t}{\lambda }\;\text{and}\;|\lambda |\to \infty ,$$

this last result (1.11) yields

1.12 $$\;\begin{array}{cc} \;\overset{\infty }{\underset{n=0}{\sum }}\Phi (z,s+n,a)\frac{t^{n}}{n!} & =\overset{\infty }{\underset{k=0}{\sum }}\frac{z^{k}}{{\left(k+a\right)}^{s}}\;\exp\left(\frac{t}{k+a}\right)\\ =:\varphi (z,t;s,a)\;(|t|<\infty ). \end{array}$$

Wilton (1922/1923) applied the expansion formula (1.9) in order to rederive Burnside’s formula (Erdélyi *et al.*^1953^, p. 48, Equation 1.18 (11)) for the sum of a series involving the Hurwitz (or generalized) Zeta function *ζ*(*s*, *a*). Srivastava (see, for details, Srivastava (1988a;1988b)), on the other hand, made use of such expansion formulas as (1.9) and (1.10) as well as the obvious special case of (1.9) when *a*=1 for finding the sums of various classes of series involving the Riemann Zeta function *ζ*(*s*) and the Hurwitz (or generalized) Zeta function *ζ*(*s*, *a*) (see also Srivastava and Choi ((2001), Chapter 3) and (Srivastava and Choi 2012), Chapter 3).

Various results for the generating functions *ϑ*_*λ*_(*z*, *t*;*s*, *a*) and *φ*(*z*, *t*;*s*, *a*), which are defined by (1.11) and (1.12), respectively, were given recently by Bin-Saad (2007, p. 46, Equations (5.1) to (5.4)) who also considered each of the following *truncated* forms of these generating functions:

1.13 $$\begin{array}{ll} \;\vartheta_{\lambda }^{\left(0,r\right)}(z,t;s,a) & :=\overset{r}{\underset{k=0}{\sum }}\frac{z^{k}}{{\left(k+a\right)}^{s-\lambda }{\left(k+a-t\right)}^{\lambda }}\;(r\in N_{0}),\; \end{array}$$

1.14 $$\begin{array}{ll} \;\vartheta_{\lambda }^{\left(r+1,\infty \right)}(z,t;s,a) & :=\overset{\infty }{\underset{k=r+1}{\sum }}\frac{z^{k}}{{\left(k+a\right)}^{s-\lambda }{\left(k+a-t\right)}^{\lambda }}\;(r\in N_{0}),\; \end{array}$$

1.15 $$\begin{array}{ll} \;\varphi^{\left(0,r\right)}(z,t;s,a) & :=\overset{r}{\underset{k=0}{\sum }}\frac{z^{k}}{{\left(k+a\right)}^{s}}\;\exp\left(\frac{t}{k+a}\right)\;(r\in N_{0})\; \end{array}$$

and

1.16 $$\;\begin{array}{c} \varphi^{\left(r+1,\infty \right)}(z,t;s,a):=\overset{\infty }{\underset{k=r+1}{\sum }}\frac{z^{k}}{{\left(k+a\right)}^{s}}\;\exp\left(\frac{t}{k+a}\right)\;(r\in N_{0}), \end{array}$$

so that, obviously, we have

1.17 $$\vartheta_{\lambda }^{\left(0,r\right)}(z,t;s,a)+\vartheta_{\lambda }^{\left(r+1,\infty \right)}(z,t;s,a)=\vartheta_{\lambda }(z,t;s,a)$$

and

1.18 $$\varphi^{\left(0,r\right)}(z,t;s,a)+\varphi^{\left(r+1,\infty \right)}(z,t;s,a)=\varphi (z,t;s,a).$$

For the Riemann Zeta function *ζ*(*s*), the special case of each of the generating functions *ϑ*_*λ*_(*z*, *t*;*s*, *a*) and *φ*(*z*, *t*;*s*, *a*) in (1.11) and (1.12) when *z* = *a* = 1 was investigated by Katsurada (1997). Subsequently, various results involving the generating functions *ϑ*_*λ*_(*z*, *t*;*s*, *a*) and *φ*(*z*, *t*;*s*, *a*) defined by (1.11) and (1.12), respectively, together with their such partial sums as those given by (1.13) to (1.16), were derived by Bin-Saad (2007) (see also the more recent sequels to (Bin-Saad 2007) and (Katsurada 1997) by Gupta and Kumari (2011) *and* by Saxena *et al.* (2011a).

Our main objective in this paper is to investigate, in a rather systematic manner, much more general families of generating functions and their partial sums than those associated with the generating functions *ϑ*_*λ*_(*z*, *t*;*s*, *a*) and *φ*(*z*, *t*;*s*, *a*) defined by (1.11) and (1.12), respectively. We also show the hitherto unnoticed fact that the so-called *τ*-generalized Riemann Zeta function, which happens to be the main subject of investigation by Gupta and Kumari (2011) and by Saxena *et al.* (2011a), is simply a seemingly trivial notational variation of the familiar general Hurwitz-Lerch Zeta function Φ(*z*, *s*, *a*) defined by (1.1). Finally, we present a sum-integral representation formula for the general family of the extended Hurwitz-Lerch Zeta functions.

## Families of the extended Hurwitz-Lerch Zeta functions and related special functions

We begin this section by recalling the following sum-integral representation given by Yen *et al.* ((2002), p. 100, Theorem) for the Hurwitz (or generalized) Zeta function *ζ*(*s*, *a*) defined by (1.3):

2.1 $$\begin{array}{l} \zeta (s,a)=\frac{1}{\Gamma (s)}\overset{k-1}{\underset{j=0}{\sum }}\int_{0}^{\infty }\frac{t^{s-1}\;e^{-(a+j)t}}{1-e^{-\text{kt}}}\;dt\;\\ \left(k\in N;\;ℜ(s)>1;\;ℜ(a)>0\right), \end{array}$$

which, for *k* = 2, was derived earlier by Nishimoto *et al.*((2002), p. 94, Theorem 4). The following straightforward generalization of the sum-integral representation (2.1) involving the familiar general Hurwitz-Lerch Zeta function Φ(*z*, *s*, *a*) defined by (1.1) was given by Lin and Srivastava (2004, p. 727, Equation (7)):

2.2 $$\begin{array}{l} \Phi (z,s,a)=\frac{1}{\Gamma (s)}\overset{k-1}{\underset{j=0}{\sum }}z^{j}\;\int_{0}^{\infty }\frac{t^{s-1}\;e^{-(a+j)t}}{1-z^{k}e^{-\text{kt}}}\;dt\;\\ \left(k\in N;\;ℜ(a)>0;\;ℜ(s)>0\;\text{when}\right.\;\;\\ \;\left.|z|≦1\;(z\neq 1);\;ℜ(s)>1\;\text{when}\;z=1\right).\; \end{array}$$

The sum-integral representations (2.1) and (1.2) led Lin and Srivastava (2004) to the introduction and investigation of an interesting generalization of the Hurwitz-Lerch Zeta function Φ(*z*, *s*, *a*) in the following form given by Lin and Srivastava ((2004), p. 727, Equation (8)):

2.3 $$\begin{array}{l} \;\Phi_{\mu ,\nu }^{\left(\rho ,\sigma \right)}(z,s,a):=\overset{\infty }{\underset{n=0}{\sum }}\frac{{\left(\mu \right)}_{\mathrm{\rho n}}}{{\left(\nu \right)}_{\mathrm{\sigma n}}}\;\frac{z^{n}}{{\left(n+a\right)}^{s}}\;\\ \;\left(\mu \in C;\;\;a,\nu \in C\setminus Z_{0}^{-};\;\;\rho ,\sigma \in R^{+};\;\;\rho <\sigma \;\text{when}\right.\;\;\\ \;s,z\in C;\;\rho =\sigma \;\;\text{and}\;\;s\in C\;\;\text{when}\;|z|<\delta :=\rho^{-\rho }\;\sigma^{\sigma };\;\;\;\\ \;\left.\rho =\sigma \;\text{and}\;ℜ(s-\mu +\nu )>1\;\text{when}\;|z|=\delta \right),\; \end{array}$$

where (*λ*)_*ν*_ denotes the Pochhammer symbol defined, in terms of the familiar Gamma function, by (1.8). Clearly, we find from the definition (2.3) that

2.4 $$\Phi_{\nu ,\nu }^{\left(\sigma ,\sigma \right)}(z,s,a)=\Phi_{\mu ,\nu }^{\left(0,0\right)}(z,s,a)=\Phi (z,s,a)$$

and

2.5 $$\begin{array}{l} \Phi_{\mu ,1}^{\left(1,1\right)}(z,s,a)=\Phi_{\mu }^{*}(z,s,a):=\overset{\infty }{\underset{n=0}{\sum }}\;\frac{{\left(\mu \right)}_{n}}{n!}\;\frac{z^{n}}{{\left(n+a\right)}^{s}}\;\\ \left(\mu \in C;\;a\in C\setminus Z_{0}^{-};\;s\in C\;\text{when}\;|z|<1;\right.\;\;\\ \left.ℜ(s-\mu )>1\;\text{when}\;|z|=1\right),\; \end{array}$$

where, as already pointed out by Lin and Srivastava (2004), $\Phi_{\mu }^{*}(z,s,a)$ is a generalization of the Hurwitz-Lerch Zeta function considered by Goyal and Laddha ((1997), p. 100, Equation (1.5)). For further results involving these classes of generalized Hurwitz-Lerch Zeta functions, see the recent works by Garg *et al.* (2006) and Lin *et al.*(2006).

A generalization of the above-defined Hurwitz-Lerch Zeta functions Φ(*z*, *s*, *a*) and $\Phi_{\mu }^{*}(z,s,a)$ was studied, in the following form, by Garg *et al.*((2008), p. 313, Equation (1.7)):

2.6 $$\begin{array}{l} \Phi_{\lambda ,\mu ;\nu }(z,s,a):=\overset{\infty }{\underset{n=0}{\sum }}\frac{{\left(\lambda \right)}_{n}{\left(\mu \right)}_{n}}{{\left(\nu \right)}_{n}\cdot n!}\;\frac{z^{n}}{{\left(n+a\right)}^{s}}\;\\ \left(\lambda ,\mu \in C;\;\nu ,a\in C\setminus Z_{0}^{-};\;s\in C\;\text{when}\;|z|<1;\right.\;\;\\ \left.ℜ(s+\nu -\lambda -\mu )>1\;\text{when}\;|z|=1\right).\; \end{array}$$

Various integral representations and two-sided bounding inequalities for Φ_*λ*, *μ*;*ν*_(*z*, *s*, *a*) can be found in the works by Garg *et al.* (2008) and [Jankov *et al.*(2011)], respectively. These latter authors [Jankov *et al.*(2011)] also considered the function Φ_*λ*, *μ*;*ν*_(*z*, *s*, *a*) as a special kind of Mathieu type (***a***, ***λ***)-series.

If we compare the definitions (2.3) and (2.6), we can easily observe that the function Φ_*λ*, *μ*;*ν*_(*z*, *s*, *a*) studied by Garg *et al.*(2008) does *not* provide a generalization of the function $\Phi_{\mu ,\nu }^{\left(\rho ,\sigma \right)}(z,s,a)$ which was introduced earlier by Lin and Srivastava (2004). Indeed, for *λ* = 1, the function Φ_*λ*, *μ*;*ν*_(*z*, *s*, *a*) coincides with a *special* case of the function $\Phi_{\mu ,\nu }^{\left(\rho ,\sigma \right)}(z,s,a)$ when *ρ* = *σ* = 1, that is,

$$\Phi_{1,\mu ;\nu }(z,s,a)=\Phi_{\mu ,\nu }^{\left(1,1\right)}(z,s,a).$$

Next, for the *Riemann-Liouville fractional derivative operator*$D_{z}^{\mu }$ defined by (see, for example, Erdélyi *et al.*((1954), p. 181), Samko *et al.*(1993) and (Kilbas et al. 2006, p. 70 *et seq.*))

2.7 $$\;\begin{array}{l} D_{z}^{\mu }\left\{f\left(z\right)\right\}\\ \;\;:=\left\{\;\;\begin{array}{c} \frac{1}{\Gamma \left(-\mu \right)}\overset{z}{\underset{0}{\int }}{\left(z-t\right)}^{-\mu -1}f\left(t\right)\text{dt}\;\left(ℜ\left(\mu \right)<0\right)\\ \frac{d^{m}}{dz^{m}}\left\{D_{z}^{\mu -m}\left\{f\left(z\right)\right\}\right\}\;\left(m-1≦ℜ\left(\mu \right)<m\left(m\in N\right)\right), \end{array}\right. \end{array}$$

the following formula is well-known:

2.8 $$D_{z}^{\mu }\left\{z^{\lambda }\right\}=\frac{\Gamma \left(\lambda +1\right)}{\Gamma \left(\lambda -\mu +1\right)}z^{\lambda -\mu }\;\left(ℜ\left(\lambda \right)>-1\right),$$

which, by virtue of the definitions (1.1) and (2.3), yields the following fractional derivative formula for the generalized Hurwitz-Lerch Zeta function $\Phi_{\mu ,\nu }^{\left(\rho ,\sigma \right)}\left(z,s,a\right)$*withρ* = *σ* [Lin and Srivastava ((2004), p. 730, Equation (24))]:

2.9 $$\begin{array}{ll} \;D_{z}^{\mu -\nu }\left\{z^{\mu -1}\Phi \left(z^{\sigma },s,a\right)\right\} & =\frac{\Gamma \left(\mu \right)}{\Gamma \left(\nu \right)}z^{\nu -1}\Phi_{\mu ,\nu }^{\left(\sigma ,\sigma \right)}\left(z^{\sigma },s,a\right)\;\\ \left(ℜ\left(\mu \right)>0;\sigma \in R^{+}\right).\; \end{array}$$

In its particular case when *ν* = *σ* = 1, the fractional derivative formula (2.9) would reduce at once to the following form:

2.10 $$\;\begin{array}{c} \Phi_{\mu }^{*}\left(z,s,a\right)=\frac{1}{\Gamma \left(\mu \right)}D_{z}^{\mu -1}\left\{z^{\mu -1}\Phi \left(z,s,a\right)\right\}\;\left(ℜ(\mu )>0\right), \end{array}$$

which (as already remarked by Lin and Srivastava (2004), p. 730) exhibits the interesting (and useful) fact that $\Phi_{\mu }^{*}(z,s,a)$ is essentially a Riemann-Liouville fractional derivative of the classical Hurwitz-Lerch function Φ(*z*, *s*, *a*). Moreover, it is easily deduced from the fractional derivative formula (2.8) that

2.11 $$\begin{array}{ll} \Phi_{\lambda ,\mu ;\nu }(z,s,a) & =\frac{\Gamma (\nu )}{\Gamma (\lambda )}\;z^{1-\lambda }\;D_{z}^{\lambda -\nu }\left\{z^{\lambda -1}\;\Phi_{\mu }^{*}(z,s,a)\right\}\;\\ =\frac{\Gamma (\nu )}{\Gamma (\lambda )\Gamma (\mu )}\;z^{1-\lambda }\;D_{z}^{\lambda -\nu }\;\;\\ \;\cdot \left\{z^{\lambda -1}\;D_{z}^{\mu -1}\left\{z^{\mu -1}\;\Phi_{\mu }(z,s,a)\right\}\right\},\; \end{array}$$

which (as observed recently by Srivastava *et al.* (2011), pp. 490–491) exhibits the fact that the function Φ_*λ*, *μ*;*ν*_(*z*, *s*, *a*) studied by Garg *et al.* (2008) is essentially a consequence of the classical Hurwitz-Lerch Zeta function Φ(*z*, *s*, *a*) when we apply the Riemann-Liouville fractional derivative operator $D_{z}^{\mu }$*two times* as indicated above in (2.11). The interested reader may be referred also to many other explicit representations for $\Phi_{\mu }^{*}(z,s,a)$ and $\Phi_{\mu ,\nu }^{\left(\rho ,\sigma \right)}(z,s,a)$, which were proven by Lin and Srivastava (2004), including (for example) a potentially useful Eulerian integral representation of the first kind [Lin and Srivastava ((2004), p. 731, Equation (28))].

It should be remarked here that a multiple (or, simply, *n*-dimentional) Hurwitz-Lerch Zeta function Φ_*n*_(*z*, *s*, *a*) was studied recently by Choi *et al.* ((2008), p. 66, Eq. (6)). On the other hand, Răducanu and Srivastava (see (Răducanu and Srivastava 2007), the references cited therein *as well as* many sequels thereto) made use of the Hurwitz-Lerch Zeta function Φ(*z*, *s*, *a*) in defining a certain linear convolution operator in their systematic investigation of various analytic function classes in *Geometric Function Theory* in *Complex Analysis*. Furthermore, Gupta *et al.* (2008) revisited the study of the familiar Hurwitz-Lerch Zeta distribution by investigating its structural properties, reliability properties and statistical inference. These investigations by Gupta *et al.* (2008) and others (see, for example, (Srivastava 2000), Srivastava and Choi (2001) and Srivastava *et al.* (2010); see also Saxena *et al.* (2011b) and Srivastava *et al.* (2011)), fruitfully using the Hurwitz-Lerch Zeta function Φ(*z*, *s*, *a*) and some of its above-mentioned generalizations, have led eventually to the following definition a family of the extended (multi-parameter) Hurwitz-Lerch Zeta functions by Srivastava *et al.* (2011).

### Definition 1

(Srivastava *et al.* (2011)). The family of the extended (multi-parameter) Hurwitz-Lerch Zeta functions

$$\Phi_{\lambda_{1},\cdots \;,\lambda_{p};\mu_{1},\cdots \;,\mu_{q}}^{\left(\rho_{1},\cdots \;,\rho_{p},\sigma_{1},\cdots \;,\sigma_{q}\right)}(z,s,a)$$

is defined by

2.12 $$\begin{array}{l} \;\Phi_{\lambda_{1},\cdots \;,\lambda_{p};\mu_{1},\cdots \;,\mu_{q}}^{\left(\rho_{1},\cdots \;,\rho_{p},\sigma_{1},\cdots \;,\sigma_{q}\right)}(z,s,a):=\overset{\infty }{\underset{n=0}{\sum }}\frac{\overset{p}{\underset{j=1}{\prod }}{\left(\lambda_{j}\right)}_{n\rho_{j}}}{n!\;\overset{q}{\underset{j=1}{\prod }}{\left(\mu_{j}\right)}_{n\sigma_{j}}}\;\frac{z^{n}}{{\left(n+a\right)}^{s}}\;\\ \;\;\;\;\;\;\;\;=:\overset{\infty }{\underset{n=0}{\sum }}\Xi_{n}\;\frac{z^{n}}{{\left(n+a\right)}^{s}}\;\\ \;\left(p,q\in N_{0};\;\lambda_{j}\in C\;(j\;=\;1,\cdots \;,p);\;a,\mu_{j}\in C\setminus Z_{0}^{-}\;(j\;=\;1,\cdots \;,q);\right.\;\\ \;\rho_{j},\sigma_{k}\in R^{+}\;(j=1,\cdots \;,p;\;k=1,\cdots \;,q);\Delta >-1\;\text{when}\;\;\\ \;s,z\in C;\;\Delta =-1\;\text{and}\;s\in C\;\text{when}\;|z|<\nabla^{*};\;\Delta =-1\;\;\\ \;\left.\text{and}\;ℜ(\Xi )>\frac{1}{2}\;\text{when}\;|z|=\nabla^{*}\right)\; \end{array}$$

where the sequence ${\left\{\Xi_{n}\right\}}_{n\in N_{0}}$ of the coefficients in the definition (2.12) is given, for latter convenience, by

2.13 $$\Xi_{n}:=\frac{\overset{p}{\underset{j=1}{\prod }}{\left(\lambda_{j}\right)}_{n\rho_{j}}}{n!\;\overset{q}{\underset{j=1}{\prod }}{\left(\mu_{j}\right)}_{n\sigma_{j}}}\;(n\in N_{0}),$$

${\left(\lambda \right)}_{\nu }\;\;(\lambda ,\nu \in C)$ denotes the Pochhammer symbol given by (1.8) and

2.14 $$\begin{array}{c} \Delta :=\overset{q}{\underset{j=1}{\sum }}\sigma_{j}-\overset{p}{\underset{j=1}{\sum }}\rho_{j},\;\;\\ \Xi :=s+\overset{q}{\underset{j=1}{\sum }}\mu_{j}-\overset{p}{\underset{j=1}{\sum }}\lambda_{j}+\frac{p-q}{2} \end{array}$$

and

$$\begin{array}{c} \nabla^{*}:=\left(\overset{p}{\underset{j=1}{\prod }}\rho_{j}^{-\rho_{j}}\right)\cdot \left(\overset{q}{\underset{j=1}{\prod }}\sigma_{j}^{\sigma_{j}}\right). \end{array}$$

In order to derive *direct* relationships of the family of the extended (multi-parameter) Hurwitz-Lerch Zeta functions

$$\Phi_{\lambda_{1},\cdots \;,\lambda_{p};\mu_{1},\cdots \;,\mu_{q}}^{\left(\rho_{1},\cdots \;,\rho_{p},\sigma_{1},\cdots \;,\sigma_{q}\right)}(z,s,a)$$

defined by (2.12) with several other relatively more familiar special functions, we need each of the following definitions.

### Definition 2

The Fox-Wright function ${}_{p}\Psi_{q}\;\;(p,q\in N_{0})$ or ${}_{p}\Psi_{q}^{*}\;\;(p,q\in N_{0})$, which is a *further* generalization of the familiar generalized hypergeometric function ${}_{p}F_{q}\;\;(p,q\in N_{0})$, with *p* numerator parameters *a*_1_, ⋯, *a*_*p*_ and *q* denominator parameters *b*_1_, ⋯, *b*_*q*_ such that

$$\begin{array}{l} a_{j}\in C\;\;(j=1,\;\cdots \;,p) \end{array}$$

and

$$\begin{array}{l} b_{j}\in C\setminus Z_{0}^{-}\;\;(j=1,\;\cdots \;,q), \end{array}$$

defined by (see, for details, (Erdélyi *et al.*^1953^, p. 183) and (Choi *et al.*^1985^, p. 21); see also (Kilbas *et al.*^2006^, p. 56), (Choi et al. 2010, p. 30) and (Srivastava *et al.*^1982^, p. 19))

2.15 $$\begin{array}{ll} {}_{p}\Psi_{q}^{*} & \left[\begin{array}{c} \left(a_{1},A_{1}\right),\;\cdots \;,\left(a_{p},A_{p}\right);\\ \left(b_{1},B_{1}\right),\;\cdots \;,\left(b_{q},B_{q}\right); \end{array}z\right]\;\\ :=\overset{\infty }{\underset{n=0}{\sum }}\frac{{\left(a_{1}\right)}_{A_{1}n}\;\cdots {\left(a_{p}\right)}_{A_{p}n}}{{\left(b_{1}\right)}_{B_{1}n}\;\cdots {\left(b_{q}\right)}_{B_{q}n}}\frac{z^{n}}{n!}\;\\ =\frac{\Gamma \left(b_{1}\right)\;\cdots \Gamma \left(b_{q}\right)}{\Gamma \left(a_{1}\right)\;\cdots \Gamma \left(a_{p}\right)}\;{}_{p}\Psi_{q}\left[\begin{array}{c} \left(a_{1},A_{1}\right),\;\cdots \;,\left(a_{p},A_{p}\right);\\ \left(b_{1},B_{1}\right),\;\cdots \;,\left(b_{q},B_{q}\right); \end{array}z\right]\; \end{array}$$

$$\begin{array}{l} \left(A_{j}>0\;\left(j=1,\;\cdots \;,p\right);B_{j}>0\;\right.\;\\ \;\left.\left(j=1,\;\cdots \;,q\right);1+\overset{q}{\underset{j=1}{\sum }}B_{j}-\overset{p}{\underset{j=1}{\sum }}A_{j}≧0\right),\; \end{array}$$

where the equality in the convergence condition holds true for suitably bounded values of |*z*| given by

2.16 $$|z|<\nabla :=\left(\overset{p}{\underset{j=1}{\prod }}A_{j}^{-A_{j}}\right)\cdot \left(\overset{q}{\underset{j=1}{\prod }}B_{j}^{B_{j}}\right).$$

In the particular case when

$$A_{j}=B_{k}=1\;(j=1,\;\cdots \;,p;\;\;k=1,\;\cdots \;,q),$$

we have the following relationship (see, for details, (Choi *et al.*^1985^, p. 21)):

2.17 $$\;\begin{array}{l} {}_{p}\Psi_{q}^{*}\left[\begin{array}{c} \left(a_{1},1\right),\;\cdots \;,\left(a_{p},1\right);\\ \left(b_{1},1\right),\;\cdots \;,\left(b_{q},1\right); \end{array}z\right]\\ \;={}_{p}F_{q}\left[\begin{array}{c} a_{1},\;\cdots \;,a_{p};\\ b_{1},\;\cdots \;,b_{q}; \end{array}z\right]\\ \;=\frac{\Gamma \left(b_{1}\right)\;\cdots \Gamma \left(b_{q}\right)}{\Gamma \left(a_{1}\right)\;\cdots \Gamma \left(a_{p}\right)}\;{}_{p}\Psi_{q}\left[\begin{array}{c} \left(a_{1},1\right),\;\cdots \;,\left(a_{p},1\right);\\ \left(b_{1},1\right),\;\cdots \;,\left(b_{q},1\right); \end{array}z\right], \end{array}$$

in terms of the generalized hypergeometric function ${}_{p}F_{q}\;\;(p,q\in N_{0})$.

### Definition 3

An attempt to derive Feynman integrals in two different ways, which arise in perturbation calculations of the equilibrium properties of a magnetic mode of phase transitions, led naturally to the following generalization of Fox’s *H*-function (Inayat-Hussain 1987b, p. 4126) (see also (Buschman and Srivastava 1990) and (Inayat-Hussain 1987a)):

2.18 $$\begin{array}{ll} \bar{H}(z) & ={\bar{H}}_{p,q}^{m,n}[z]\;\\ ={\bar{H}}_{p,q}^{m,n}\left[\;z\;\left|\;\begin{array}{c} {\left(a_{j},A_{j};\alpha_{j}\right)}_{j=1}^{n},{\left(a_{j},A_{j}\right)}_{j=n+1}^{p}\\ {b_{j},B_{j})}_{j=1}^{m},{\left(b_{j},B_{j};\beta_{j}\right)}_{j=m+1}^{q} \end{array}\;\right]\right.\\ :=\frac{1}{2\pi i}\int_{L}\chi (s)z^{s}\;ds\; \end{array}$$

$$\begin{array}{l} \left(z\neq 0;\;i=\sqrt{-1};\;\chi (s)\right.\;\\ \;\left.:=\frac{\overset{m}{\underset{j=1}{\prod }}\Gamma (b_{j}-B_{j}s)\cdot \overset{n}{\underset{j=1}{\prod }}{\left\{\Gamma (1-a_{j}+A_{j}s)\right\}}^{\alpha_{j}}}{\overset{p}{\underset{j=n+1}{\prod }}\Gamma (a_{j}-A_{j}s)\cdot \overset{q}{\underset{j=m+1}{\prod }}{\left\{\Gamma (1-b_{j}+B_{j}s)\right\}}^{\beta_{j}}}\right),\; \end{array}$$

which contains *fractional* powers of some of the Gamma functions involved. Here, and in what follows, the parameters

$$\;A_{j}>0\;\;(j=1,\cdots \;,p)\;\text{and}\;B_{j}>0\;\;(j=1,\cdots \;,q),$$

the exponents

$$\alpha_{j}\;(j=1,\cdots \;,n)\;\text{and}\;\beta_{j}\;(j=m+1,\cdots \;,q)$$

can take on noninteger values, and $L=L_{\left(i\tau ;\infty \right)}$ is a Mellin-Barnes type contour starting at the point *τ* − i*∞* and terminating at the point *τ* + i*∞*(τ∈R) with the usual indentations to separate one set of poles from the other set of poles. The sufficient condition for the absolute convergence of the contour integral in (2.18) was established as follows by Buschman and Srivastava ((1990), p. 4708):

2.19 $$\Lambda :=\overset{m}{\underset{j=1}{\sum }}B_{j}+\overset{n}{\underset{j=1}{\sum }}|\alpha_{j}|A_{j}-\overset{q}{\underset{j=m+1}{\sum }}|\beta_{j}|B_{j}-\overset{p}{\underset{j=n+1}{\sum }}A_{j}>0,$$

which provides exponential decay of the integrand in (2.18) and the region of absolute convergence of the contour integral in (2.18) is given by

2.20 $$|\arg(z)|<\frac{1}{2}\;\pi \Lambda ,$$

where *Λ* is defined by (2.19).

## Remark 1

If we set

$$\;\begin{array}{c} s=0,\;\;p\mapsto p+1\;\left(\rho_{1}=\cdots =\rho_{p}=1;\;\;\lambda_{p+1}=\rho_{p+1}=1\right) \end{array}$$

and

$$\;\begin{array}{c} q\mapsto q+1\;\left(\sigma_{1}=\cdots =\sigma_{q}=1;\;\;\mu_{q+1}=\beta ;\;\;\sigma_{q+1}=\alpha \right), \end{array}$$

then (2.12) reduces to the following generalized *M*-series which was recently introduced by Sharma and Jain (2009) (see also an earlier paper by Sharma (2008) for the *special* case when *β*=1):

2.21 $$\begin{array}{ll} {}_{p}{\overset{\alpha ,\beta }{M}}_{q} & \left(a_{1},\cdots \;,a_{p};b_{1},\cdots \;,b_{q};z\right)\\ :=\overset{\infty }{\underset{k=0}{\sum }}\frac{{\left(a_{1}\right)}_{k}\cdots {\left(a_{p}\right)}_{k}}{{\left(b_{1}\right)}_{k}\cdots {\left(b_{q}\right)}_{k}}\;\frac{z^{k}}{\Gamma (\mathrm{\alpha k}+\beta )}\\ =\frac{1}{\Gamma (\beta )}\;{}_{p+1}\Psi_{q+1}^{*}\left[\begin{array}{c} \left(a_{1},1\right),\;\cdots \;,\left(a_{p},1\right),(1,1);\\ \left(b_{1},1\right),\;\cdots \;,\left(b_{q},1\right),(\beta ,\alpha ); \end{array}z\right]\;,\; \end{array}$$

in which the last relationship exhibits the fact that the so-called generalized *M*-series is indeed an *obvious* special case of the Fox-Wright function ${}_{p}\Psi_{q}^{*}$ defined by (2.15) (see also (Saxana 2009)). Similarly, for the generalized Mittag-Leffler function considered by Kilbas *et al.* (2002), we have

2.22 $$\;\begin{array}{c} E_{\rho }\left[\left(\beta_{1},\eta_{1}\right),\cdots \;,\left(\beta_{q},\eta_{q}\right);z\right]\\ \;:=\overset{\infty }{\underset{k=0}{\sum }}\frac{{\left(\rho \right)}_{k}}{\overset{q}{\underset{j=1}{\prod }}\Gamma \left(\eta_{j}k+\beta_{j}\right)}\\ \;=\frac{1}{\Gamma (\rho )}\;{}_{1}\Psi_{q}\left[\begin{array}{r} \left(\rho ,1\right);\\ \left(\beta_{1},\eta_{1}\right),\;\cdots \;,\left(\beta_{q},\eta_{q}\right); \end{array}z\right], \end{array}$$

## Remark 2

The following $\bar{H}$-function representation can be applied in order to derive various properties of the extended Hurwitz-Lerch Zeta function

$$\Phi_{\lambda_{1},\cdots \;,\lambda_{p};\mu_{1},\cdots \;,\mu_{q}}^{\left(\rho_{1},\cdots \;,\rho_{p},\sigma_{1},\cdots \;,\sigma_{q}\right)}(z,s,a)$$

from those of the $\bar{H}$-function (see, for details, Srivastava *et al.* ((2011), p. 504, Theorem 8)):

2.23 $$\;\begin{array}{l} \Phi_{\lambda_{1},\cdots \;,\lambda_{p};\mu_{1},\cdots \;,\mu_{q}}^{\left(\rho_{1},\cdots \;,\rho_{p},\sigma_{1},\cdots \;,\sigma_{q}\right)}(z,s,a)\\ =\frac{\overset{q}{\underset{j=1}{\prod }}\Gamma \left(\mu_{j}\right)}{\overset{p}{\underset{j=1}{\prod }}\Gamma \left(\lambda_{j}\right)}\;{\bar{H}}_{p+1,q+2}^{1,p+1}\\ \;\;\left[-z\left|\begin{array}{c} \left(1-\lambda_{1},\rho_{1};1),\cdots \;,(1-\lambda_{p},\rho_{p};1),(1-a,1;s\right)\\ \left(0,1),(1-\mu_{1},\sigma_{1};1),\cdots \;,(1-\mu_{q},\sigma_{q};1),(-a,1;s\right) \end{array}\right]\right.\\ =\frac{\overset{q}{\underset{j=1}{\prod }}\;\Gamma \;\left(\mu_{j}\right)}{\overset{p}{\underset{j=1}{\prod }}\;\Gamma \;\left(\lambda_{j}\right)}\;\cdot \;\frac{1}{2\pi i}\;\;\int_{ℒ}\;\frac{\Gamma (-\xi ){\left\{\Gamma (\xi \;+\;a)\right\}}^{s}\;\overset{p}{\underset{j=1}{\prod }}\;\Gamma \left(\lambda_{j}\;+\;\rho_{j}\xi \right)}{{\left\{\Gamma (\xi \;+\;a\;+\;1)\right\}}^{s}\;\overset{q}{\underset{j=1}{\prod }}\;\Gamma \left(\mu_{j}\;+\;\sigma_{j}\xi \right)}\;{\left(-z\right)}^{\xi }d\xi \\ \left(|\arg(-z)|<\pi \right), \end{array}$$

the path of integrationL in the last member of (2.23) being a Mellin-Barnes type contour in the complex *ξ*-plane, which starts at the point −i*∞* and terminates at the point i*∞* with indentations, if necessary, in such a manner as to separate the poles of *Γ*(−*ξ*) from the poles of *Γ*(*λ*_*j*_+*ρ*_*j*_*ξ*) (*j*=1,⋯, *p*). Thus, for example, by making use of a known fractional-calculus result due to Srivastava *et al.* ((2006), p. 97, Equation (2.4)), we readily obtain the following extension of such fractional derivative formulas as (2.9) and (2.10) [Srivastava *et al.* ((2011), p. 505, Equation (6.8))]:

2.24 $$\begin{array}{l} D_{z}^{\nu -\tau }\left\{z^{\nu -1}\;\Phi_{\lambda_{1},\cdots \;,\lambda_{p};\mu_{1},\cdots \;,\mu_{q}}^{\left(\rho_{1},\cdots \;,\rho_{p},\sigma_{1},\cdots \;,\sigma_{q}\right)}(z^{\kappa },s,a)\right\}=\frac{\overset{q}{\underset{j=1}{\prod }}\Gamma \left(\mu_{j}\right)}{\overset{p}{\underset{j=1}{\prod }}\Gamma \left(\lambda_{j}\right)}\;z^{\tau -1}\;\\ \cdot {\bar{H}}_{p+2,q+3}^{1,p+2}\left[-z^{\kappa }\left|\begin{array}{c} \left(1-\lambda_{1},\rho_{1};1),\cdots \;,(1-\lambda_{p},\rho_{p};1),(1-\nu ,\kappa ;1),(1-a,1;s\right)\\ \left(0,1),(1-\mu_{1},\sigma_{1};1),\cdots \;,(1-\mu_{q},\sigma_{q};1),(1-\tau ,\kappa ;1),(-a,1;s\right) \end{array}\right]\right.\\ \;=\frac{\Gamma (\nu )}{\Gamma (\tau )}\;z^{\tau -1}\;\Phi_{\lambda_{1},\cdots \;,\lambda_{p},\nu ;\mu_{1},\cdots \;,\mu_{q},\tau }^{\left(\rho_{1},\cdots \;,\rho_{p},\kappa ,\sigma_{1},\cdots \;,\sigma_{q},\kappa \right)}(z^{\kappa },s,a)\;\left(ℜ(\nu )>0;\;\kappa >0\right).\; \end{array}$$

## Generating relations associated with the extended Hurwitz-Lerch Zeta function

In this section, we first introduce the following generating functions and their partial sums involving the extended Hurwitz-Lerch Zeta function

$$\Phi_{\lambda_{1},\cdots \;,\lambda_{p};\mu_{1},\cdots \;,\mu_{q}}^{\left(\rho_{1},\cdots \;,\rho_{p},\sigma_{1},\cdots \;,\sigma_{q}\right)}(z,s,a)$$

defined by (2.12). Indeed, as a generalization of the generating functions (1.9) and (1.10), we have

3.1 $$\;\begin{array}{l} \Phi_{\lambda_{1},\cdots \;,\lambda_{p};\mu_{1},\cdots \;,\mu_{q}}^{\left(\rho_{1},\cdots \;,\rho_{p},\sigma_{1},\cdots \;,\sigma_{q}\right)}(z,s,a-t)\\ \;=\overset{\infty }{\underset{n=0}{\sum }}\frac{{\left(s\right)}_{n}}{n!}\;\Phi_{\lambda_{1},\cdots \;,\lambda_{p};\mu_{1},\cdots \;,\mu_{q}}^{\left(\rho_{1},\cdots \;,\rho_{p},\sigma_{1},\cdots \;,\sigma_{q}\right)}(z,s+n,a)t^{n}\;(|t|<|a|), \end{array}$$

which can easily be put in the following more general form:

3.2 $$\;\begin{array}{l} \overset{\infty }{\underset{n=0}{\sum }}\frac{{\left(\lambda \right)}_{n}}{n!}\;\Phi_{\lambda_{1},\cdots \;,\lambda_{p};\mu_{1},\cdots \;,\mu_{q}}^{\left(\rho_{1},\cdots \;,\rho_{p},\sigma_{1},\cdots \;,\sigma_{q}\right)}(z,s+n,a)t^{n}\\ \;\;=\overset{\infty }{\underset{k=0}{\sum }}\;\frac{\Xi_{k}\;z^{k}}{{\left(k+a\right)}^{s-\lambda }{\left(k+a-t\right)}^{\lambda }}=:\Omega_{\lambda }(z,t;s,a)\;(|t|<|a|), \end{array}$$

where the sequence ${\left\{\Xi_{n}\right\}}_{n\in N_{0}}$ of the coefficients in (2.12) is given by (2.13). This last generating function (3.2) would reduce immediately to the expansion formula (4.1) in its special case when *λ* = *s*. Furthermore, in its limit case when

$$t\mapsto \frac{t}{\lambda }\;\text{and}\;|\lambda |\to \infty ,$$

the generating function (3.2) yields

3.3 $$\;\begin{array}{l} \overset{\infty }{\underset{n=0}{\sum }}\Phi_{\lambda_{1},\cdots \;,\lambda_{p};\mu_{1},\cdots \;,\mu_{q}}^{\left(\rho_{1},\cdots \;,\rho_{p},\sigma_{1},\cdots \;,\sigma_{q}\right)}(z,s+n,a)\;\frac{t^{n}}{n!}\\ \;=\overset{\infty }{\underset{k=0}{\sum }}\frac{\Xi_{k}\;z^{k}}{{\left(k+a\right)}^{s}}\;\exp\left(\frac{t}{k+a}\right)=:\Theta (z,t;s,a)\;(|t|<\infty ), \end{array}$$

where the sequence ${\left\{\Xi_{n}\right\}}_{n\in N_{0}}$ of the coefficients in (2.12) is given, as before, by (2.13).

We shall also consider each of the following *truncated* forms of the generating functions *Ω*_*λ*_(*z*, *t*;*s*, *a*) and *Θ*(*z*, *t*;*s*, *a*) in (3.2) and (3.3), respectively:

3.4 $$\begin{array}{ll} \;\Omega_{\lambda }^{\left(0,r\right)}(z,t;s,a) & :=\overset{r}{\underset{k=0}{\sum }}\frac{\Xi_{k}\;z^{k}}{{\left(k+a\right)}^{s-\lambda }{\left(k+a-t\right)}^{\lambda }}\;(r\in N_{0}),\; \end{array}$$

3.5 $$\begin{array}{ll} \;\Omega_{\lambda }^{\left(r+1,\infty \right)}(z,t;s,a) & :=\overset{\infty }{\underset{k=r+1}{\sum }}\frac{\Xi_{k}\;z^{k}}{{\left(k+a\right)}^{s-\lambda }{\left(k+a-t\right)}^{\lambda }}\;(r\in N_{0}),\; \end{array}$$

3.6 $$\begin{array}{ll} \;\Theta^{\left(0,r\right)}(z,t;s,a) & :=\overset{r}{\underset{k=0}{\sum }}\frac{\Xi_{k}\;z^{k}}{{\left(k+a\right)}^{s}}\;\exp\left(\frac{t}{k+a}\right)\;(r\in N_{0})\; \end{array}$$

and

3.7 $$\;\begin{array}{c} \Theta^{\left(r+1,\infty \right)}(z,t;s,a):=\overset{\infty }{\underset{k=r+1}{\sum }}\frac{\Xi_{k}\;z^{k}}{{\left(k+a\right)}^{s}}\;\exp\left(\frac{t}{k+a}\right)\;(r\in N_{0}), \end{array}$$

which obviously satisfy the following decomposition formulas:

3.8 $$\Omega_{\lambda }^{\left(0,r\right)}(z,t;s,a)+\Omega_{\lambda }^{\left(r+1,\infty \right)}(z,t;s,a)=\Omega_{\lambda }(z,t;s,a)$$

and

3.9 $$\Theta^{\left(0,r\right)}(z,t;s,a)+\Theta^{\left(r+1,\infty \right)}(z,t;s,a)=\Theta (z,t;s,a).$$

Our first set of integral representations for the above-defined generating functions is contained in Theorem 1 below.

### Theorem 1

Each of the following integral representation formulas holds true:

3.10 $$\;\begin{array}{lll} \Omega_{\lambda }(z,\omega ;s,a) & = & \;\frac{1}{\Gamma (s)}\;\int_{0}^{\infty }t^{s-1}\;e^{-\text{at}}{}_{p}\Psi_{q}^{*}\\ \left[\begin{array}{c} (\lambda_{1},\rho_{1}),\cdots \;,(\lambda_{p},\rho_{p});\\ (\mu_{1},\sigma_{1}),\cdots \;,(\mu_{q},\sigma_{q}); \end{array}\;ze^{-t}\right]\\ \cdot_{1}\;F_{1}\left(\lambda ;s;\mathrm{\omega t}\right)dt\;\left(\min\{ℜ(a),ℜ(s)\}>0\right) \end{array}$$

and

3.11 $$\;\begin{array}{lll} \Theta (z,\omega ;s,a) & = & \;\frac{1}{\Gamma (s)}\;\int_{0}^{\infty }t^{s-1}\;e^{-\text{at}}{}_{p}\Psi_{q}^{*}\\ \left[\begin{array}{c} (\lambda_{1},\rho_{1}),\cdots \;,(\lambda_{p},\rho_{p});\\ (\mu_{1},\sigma_{1}),\cdots \;,(\mu_{q},\sigma_{q}); \end{array}\;ze^{-t}\right]\\ \cdot_{0}\;F_{1}\left(\bar{}\;;s;\mathrm{\omega t}\right)dt\;\;\;\left(\min\{ℜ(a),ℜ(s)\}>0\right), \end{array}$$

provided that both sides of each of the assertions (3.10) and (3.11) exist.

### Proof

For convenience, we denote byS the second member of the assertion (3.10) of Theorem 1. Then, upon expanding the functions ${}_{p}\Psi_{q}^{*}$ and _1_*F*_1_ in series forms, we find that

3.12 $$\;\begin{array}{ll} S & :=\frac{1}{\Gamma (s)}\;\int_{0}^{\infty }t^{s-1}\;e^{-\text{at}}{}_{p}\Psi_{q}^{*}\\ \;{\left[\begin{array}{c} (\lambda_{1},\rho_{1}),\cdots \;,(\lambda_{p},\rho_{p});\\ (\mu_{1},\sigma_{1}),\cdots \;,(\mu_{q},\sigma_{q}); \end{array}\;ze^{-t}\right]}_{1}F_{1}\left(\lambda ;s;\mathrm{\omega t}\right)dt\\ \;=\frac{1}{\Gamma (s)}\;\overset{\infty }{\underset{m,n=0}{\sum }}\Xi_{m}\;z^{m}\;\frac{{\left(\lambda \right)}_{n}}{{\left(s\right)}_{n}}\;\frac{\omega^{n}}{n!}\int_{0}^{\infty }t^{s+n-1}\;e^{-(a+m)t}\;dt, \end{array}$$

where the inversion of the order of integration and double summation can easily be justified by absolute convergence under the conditions stated with (3.10), Ξ_*n*_ being defined by (2.13). Now, if we evaluate the innermost integral in (3.12) by appealing to the following well-known result:

3.13 $$\int_{0}^{\infty }\;t^{\mu -1}\;e^{-\mathrm{\kappa t}}\;dt=\frac{\Gamma (\mu )}{\kappa^{\mu }}\;\left(\min\{ℜ(\kappa ),ℜ(\mu )\}>0\right),$$

we get

3.14 $$\begin{array}{lll} S & = & \;\overset{\infty }{\underset{n=0}{\sum }}\;\frac{{\left(\lambda \right)}_{n}}{n!}\;\left(\overset{\infty }{\underset{m=0}{\sum }}\;\Xi_{m}\;\frac{z^{m}}{{\left(m+a\right)}^{s+n}}\right)\omega^{n}\\ \left(\min\{ℜ(a),ℜ(s)\}>0\right), \end{array}$$

which, in light of the definitions (2.12) and (3.2), yields the left-hand side of the first assertion (3.10) of Theorem 1.

The second assertion (3.11) of Theorem 1 can be proven in a similar manner. □

### Remark 3

For *ω* = 0, each of the assertions (3.10) and (3.11) of Theorem 1 yields a known integral representation formula due to Srivastava *et al.* ((2011), p. 504, Equation (6.4)). Moreover, in their special case when

$$\omega =0\;\text{and}\;\Xi_{n}=1\;(n\in N_{0}),$$

the assertions (3.10) and (3.11) of Theorem 1 would reduce immediately to the classical integral representation (1.7) for the Hurwitz-Lerch Zeta function Φ(*z*, *s*, *a*).

The proof of Theorem 2 below would run parallel to that of Theorem 1, which we already have detailed above fairly adequately. It is based essentially upon the Hankel type contour integral in the following form (Erdélyi *et al.*^1953^, p. 14, Equation 1.16 (4)):

3.15 $$\begin{array}{lll} 2i\;\sin(\pi \nu )\Gamma (\nu ) & = & -\int_{\infty }^{\left(0+\right)}\;{\left(-t\right)}^{\nu -1}\;e^{-t}\;dt\\ \left(|\arg(-t)|≦\pi \right) \end{array}$$

or, equivalently,

3.16 $$\begin{array}{lll} \frac{1}{\Gamma (1-\nu )} & = & -\frac{1}{2\pi i}\int_{\infty }^{\left(0+\right)}\;{\left(-t\right)}^{\nu -1}\;e^{-t}\;dt\\ \left(|\arg(-t)|≦\pi \right). \end{array}$$

### Theorem 2

Each of the following Hankel type contour integral representation formulas holds true :

3.17 $$\;\begin{array}{lll} \Omega_{\lambda }(z,\omega ;s,a) & = & -\frac{\Gamma (1-s)}{2\pi i}\;\int_{\infty }^{\left(0+\right)}\;{\left(-t\right)}^{s-1}\;e^{-\text{at}}{}_{p}\Psi_{q}^{*}\\ \left[\begin{array}{c} (\lambda_{1},\rho_{1}),\cdots \;,(\lambda_{p},\rho_{p});\\ (\mu_{1},\sigma_{1}),\cdots \;,(\mu_{q},\sigma_{q}); \end{array}\;ze^{-t}\right]\\ \cdot_{1}\;F_{1}\left(\lambda ;s;\mathrm{\omega t}\right)dt\;\left(ℜ(a)>0;\;|\arg(-t)|≦\pi \right) \end{array}$$

and

3.18 $$\;\begin{array}{lll} \Theta (z,\omega ;s,a) & = & -\frac{\Gamma (1-s)}{2\pi i}\;\int_{\infty }^{\left(0+\right)}\;{\left(-t\right)}^{s-1}\;e^{-\text{at}}{}_{p}\Psi_{q}^{*}\\ \left[\begin{array}{c} (\lambda_{1},\rho_{1}),\cdots \;,(\lambda_{p},\rho_{p});\\ (\mu_{1},\sigma_{1}),\cdots \;,(\mu_{q},\sigma_{q}); \end{array}\;ze^{-t}\right]\\ \cdot_{0}\;F_{1}\left(\bar{}\;;s;\mathrm{\omega t}\right)dt\;\left(ℜ(a)>0;\;|\arg(-t)|\;≦\;\pi \right), \end{array}$$

provided that both sides of each of the assertions (3.17) and (3.18) exist.

### Remark 4

For *ω* = 0, each of the assertions (3.17) and (3.18) of Theorem 2 yields the following (*presumably new*) integral representation formula:

3.19 $$\begin{array}{l} \Phi_{\lambda_{1},\cdots \;,\lambda_{p};\mu_{1},\cdots \;,\mu_{q}}^{\left(\rho_{1},\cdots \;,\rho_{p},\sigma_{1},\cdots \;,\sigma_{q}\right)}(z,s,a)\\ \;=-\frac{\Gamma (1-s)}{2\pi i}\;\int_{\infty }^{\left(0+\right)}\;{\left(-t\right)}^{s-1}\;e^{-\text{at}}\\ \;\cdot_{p}\;\Psi_{q}^{*}\left[\begin{array}{c} (\lambda_{1},\rho_{1}),\cdots \;,(\lambda_{p},\rho_{p});\\ (\mu_{1},\sigma_{1}),\cdots \;,(\mu_{q},\sigma_{q}); \end{array}\;ze^{-t}\right]dt\\ \;\left(ℜ(a)>0;\;|\arg(-t)|≦\pi \right). \end{array}$$

Furthermore, in their special case when

$$\omega =0\;\text{and}\;\Xi_{n}=1\;(n\in N_{0}),$$

the assertions (3.17) and (3.18) of Theorem 2 would reduce to the classical Hankel type contour integral representation for the Hurwitz-Lerch Zeta function Φ(*z*, *s*, *a*) (see, for example, (, Erdélyi *et al.*^1953^, p. 28, Equation 1.11 (5)); see also (Srivastava and Choi 2012), p. 195, Equation 2.5 (8)).

Next, by making use of the following known result (see, for example, Srivastava and Manocha ((1984), p. 86, Problem 1):

3.20 $$\begin{array}{c} \int_{a}^{b}\;{\left(t-a\right)}^{\alpha -1}\;{\left(b-t\right)}^{\beta -1}\;dt={\left(b-a\right)}^{\alpha +\beta -1}\;B(\alpha ,\beta )\\ \;\left(b\neq a;\;\min\{ℜ(\alpha ),ℜ(\beta )\}>0\right), \end{array}$$

we evaluate several Eulerian Beta-function integrals involving the generating functions *Ω*_*λ*_(*z*, *t*;*s*, *a*) and *Θ*(*z*, *t*;*s*, *a*) defined by (3.2) and (3.3), respectively, *B*(*α*, *β*) being the familiar Beta function.

### Theorem 3

In terms of the sequence ${\left\{\Xi_{n}\right\}}_{n\in N_{0}}$ of the coefficients given by the definition (2.13), each of the following Eulerian Beta-function integral formulas holds true:

3.21 $$\begin{array}{l} \int_{\xi }^{\eta }\;{\left(t-\xi \right)}^{\alpha -1}\;{\left(\eta -t\right)}^{\beta -1}\;\Omega_{\lambda }\left(z,\omega {\left(t-\xi \right)}^{\gamma }\;{\left(\eta -t\right)}^{\delta };s,a\right)dt\\ \;={\left(\eta -\xi \right)}^{\alpha +\beta -1}\;B(\alpha ,\beta )\;\overset{\infty }{\underset{n=0}{\sum }}\;\Xi_{n}\;\frac{z^{n}}{{\left(n+a\right)}^{s}}\;\;{}_{3}\Psi_{1}^{*}\\ \;\;\cdot \left[\begin{array}{c} (\lambda ,1),(\alpha ,\gamma ),(\beta ,\delta );\\ (\alpha +\beta ,\gamma +\delta ); \end{array}\;\frac{\omega {\left(\eta -\xi \right)}^{\gamma +\delta }}{n+a}\right]\\ \;\;\left(\eta \neq \xi ;\;\min\{ℜ(\alpha ),ℜ(\beta )\}>0;\;\gamma ,\delta >0\right) \end{array}$$

and

3.22 $$\begin{array}{l} \int_{\xi }^{\eta }\;{\left(t-\xi \right)}^{\alpha -1}\;{\left(\eta -t\right)}^{\beta -1}\;\Theta \left(z,\omega {\left(t-\xi \right)}^{\gamma }\;{\left(\eta -t\right)}^{\delta };s,a\right)dt\\ \;={\left(\eta -\xi \right)}^{\alpha +\beta -1}\;B(\alpha ,\beta )\;\overset{\infty }{\underset{n=0}{\sum }}\;\Xi_{n}\;\frac{z^{n}}{{\left(n+a\right)}^{s}}\;{}_{2}\Psi_{1}^{*}\\ \;\;\cdot \left[\begin{array}{c} (\alpha ,\gamma ),(\beta ,\delta );\\ (\alpha +\beta ,\gamma +\delta ); \end{array}\;\frac{\omega {\left(\eta -\xi \right)}^{\gamma +\delta }}{n+a}\right]\\ \;\left(\eta \neq \xi ;\;\min\{ℜ(\alpha ),ℜ(\beta )\}>0;\;\gamma ,\delta >0\right), \end{array}$$

provided that both sides of each of the assertions (3.21) and (3.22) exist, the Fox-Wright function 00${}_{3}\Psi_{1}^{*}$ in (3.21) being tacitly interpreted as an *H*-function contained in the definition (2.18).

### Proof

Each of the assertions (3.21) and (3.22) of Theorem 3 can be proven fairly easily by appealing to the definitions (3.2) and (3.3), respectively, in conjunction with the Eulerian Beta-function integral (3.20). The details involved are being skipped here. □

### Remark 5

In addition to their relatively more familiar cases when *ξ* = *η*−1 = 0, various interesting limit cases of the integral formulas (3.21) and (3.22) asserted by Theorem 3 can be deduced by letting

$$\underset{\gamma \;↓\;0}{\lim}\;\text{or}\;\underset{\delta \;↓\;0}{\lim}.$$

Some such very specialized cases of Theorem 3 can be found in the recent works by Bin-Saad (2007), Gupta and Kumari (2011) and Saxena *et al.* (2011a).

The Eulerian Gamma-function integrals involving the generating functions *Ω*_*λ*_(*z*, *t*;*s*, *a*) and *Θ*(*z*, *t*;*s*, *a*) defined by (3.2) and (3.3), respectively, which are asserted by Theorem 4 below, can be evaluated by applying the well-known formula (3.13).

### Theorem 4

Let the function $\Phi_{\mu }^{*}(z,s,a)$ be defined by (2.5). Then, in terms of the sequence ${\left\{\Xi_{n}\right\}}_{n\in N_{0}}$ of the coefficients given by the definition (2.13), each of the following single or double Eulerian Gamma-function integral formulas holds true:

3.23 $$\begin{array}{l} \frac{1}{\Gamma (\mu )}\;\int_{0}^{\infty }\;t^{\mu -1}\;e^{-\mathrm{\kappa t}}\;\Omega_{\lambda }\left(z,\omega e^{-\mathrm{\delta t}};s,a\right)dt\\ \;\;=\delta^{-\mu }\;\overset{\infty }{\underset{n=0}{\sum }}\;\frac{\Xi_{n}z^{n}}{{\left(n+a\right)}^{s}}\;\Phi_{\lambda }^{*}\left(\frac{\omega }{n+a},\mu ,\frac{\kappa }{\delta }\right)\\ \;\left(\min\{ℜ(\kappa ),ℜ(\mu ),ℜ(\delta )\}>0\right), \end{array}$$

3.24 $$\begin{array}{l} \frac{1}{\Gamma (\mu )}\;\int_{0}^{\infty }\;t^{\mu -1}\;e^{-\mathrm{\kappa t}}\;\Theta \left(z,\mathrm{\omega t};s,a\right)dt\\ \;\;=\kappa^{-\mu }\;\Omega_{\mu }\left(z,\frac{\omega }{\kappa };s,a\right)\;\left(\min\{ℜ(\kappa ),ℜ(\mu )\}>0\right) \end{array}$$

and

3.25 $$\begin{array}{l} \frac{1}{\Gamma (\mu )\Gamma (\nu )}\;\int_{0}^{\infty }\;\int_{0}^{\infty }\;u^{\mu -1}\;v^{\nu -1}\;e^{-\mathrm{\kappa u}-\mathrm{\delta v}}\;\Theta \left(z,\mathrm{\omega u}e^{-\mathrm{\sigma v}};s,a\right)du\;dv\\ \;\;=\kappa^{-\mu }\;\sigma^{-\nu }\;\overset{\infty }{\underset{n=0}{\sum }}\;\frac{\Xi_{n}z^{n}}{{\left(n+a\right)}^{s}}\;\Phi_{\mu }^{*}\left(\frac{\omega }{\kappa (n+a)},\mu ,\frac{\delta }{\sigma }\right)\\ \;\;\left(\min\{ℜ(\kappa ),ℜ(\mu ),ℜ(\nu ),ℜ(\delta ),ℜ(\sigma )\}>0\right), \end{array}$$

provided that both sides of each of the assertions (3.23), (3.24) and (3.25) exist.

### Remark 6

Some very specialized cases of Theorem 4 when

$$\Xi_{n}=1\;\;(n\in N_{0})$$

were derived in the recent works (Bin-Saad 2007), (Gupta and Kumari 2011) and (Saxena *et al.* (2011a)).

### Remark 7

Two of the claimed integral formulas in Bin-Saad’s paper (2007, p. 42, Theorem 3.2, Equations (3.10) *and* (3.11)) can easily be shown to be *divergent*, simply because the improper integrals occurring on their left-hand sides obviously violate the required convergence conditions at their *lower* terminal *t* = 0.

We now turn toward the *truncated* forms of the generating functions *Ω*_*λ*_(*z*, *t*;*s*, *a*) and *Θ*(*z*, *t*;*s*, *a*) in (3.2) and (3.3), respectively, which are defined by (3.4) to (3.7). Indeed, by appealing appropriately to the definitions in (3.4) to (3.7) in conjunction with the Eulerian Gamma-function integral in (3.13), it is fairly straightforward to derive the integral representation formulas asserted by Theorem 5 below.

### Theorem 5

In terms of the sequence ${\left\{\Xi_{n}\right\}}_{n\in N_{0}}$ of the coefficients given by the definition (2.13), each of the following Eulerian Gamma-function integral formulas holds true:

3.26 $$\begin{array}{l} \Omega_{\lambda }^{\left(0,r\right)}(z,\omega ;s,a)\\ \;=\frac{1}{\Gamma (s)}\;\int_{0}^{\infty }\;t^{s-1}\;e^{-\text{at}}\left(\overset{r}{\underset{k=0}{\sum }}\;\Xi_{k}\;{\left(ze^{-t}\right)}^{k}\right){}_{1}F_{1}(\lambda ;s;\mathrm{\omega t})dt\\ \;\;\;\left(\min\{ℜ(s),ℜ(a)\}>0\right), \end{array}$$

3.27 $$\begin{array}{l} \Omega_{\lambda }^{\left(r+1,\infty \right)}(z,\omega ;s,a)\\ \;=\frac{1}{\Gamma (s)}\int_{0}^{\infty }\;\;t^{s-1}\;e^{-\text{at}}{\left(\overset{\infty }{\underset{k=r+1}{\sum }}\;\Xi_{k}\;{\left(ze^{-t}\right)}^{k}\right)}_{1}F_{1}(\lambda ;s;\mathrm{\omega t})dt\\ \;\;\;\left(\min\{ℜ(s),ℜ(a)\}>0\right), \end{array}$$

3.28 $$\begin{array}{l} \Theta^{\left(0,r\right)}(z,\omega ;s,a)\\ \;=\frac{1}{\Gamma (s)}\int_{0}^{\infty }\;\;t^{s-1}\;e^{-\text{at}}{\left(\overset{r}{\underset{k=0}{\sum }}\;\Xi_{k}\;{\left(ze^{-t}\right)}^{k}\right)}_{0}F_{1}(\bar{}\;;s;\mathrm{\omega t})dt\\ \;\;\;\left(\min\{ℜ(s),ℜ(a)\}>0\right) \end{array}$$

and

3.29 $$\begin{array}{l} \Theta^{\left(r+1,\infty \right)}(z,\omega ;s,a)\\ \;=\frac{1}{\Gamma (s)}\int_{0}^{\infty }\;\;t^{s-1}\;e^{-\text{at}}\left(\overset{\infty }{\underset{k=r+1}{\sum }}\;\Xi_{k}\;{\left(ze^{-t}\right)}^{k}\right){}_{0}F_{1}(\bar{};s;\mathrm{\omega t})dt\\ \;\;\;\left(\min\{ℜ(s),ℜ(a)\}>0\right), \end{array}$$

provided that both sides of each of the assertions (3.26) to (3.29) exist.

### Remark 8

Several specialized cases of Theorem 5 when

$$\Xi_{n}=1\;\;(n\in N_{0})$$

can be found in the recent works (Bin-Saad 2007), (Gupta and Kumari 2011) and ( (Saxena *et al.*2011a)).

It is not difficult to derive various other properties and results involving the generating functions *Ω*_*λ*_(*z*, *t*;*s*, *a*) and *Θ*(*z*, *t*;*s*, *a*) in (3.2) and (3.3), respectively, as well as their *truncated* forms which are defined by (3.4) to (3.7). For example, by applying the definition (3.2) in conjunction with the definition (2.15), it is easy to derive the following general form of the generating relations asserted by (for example) Bin-Saad (2007, p. 44, Theorem 4.2):

3.30 $$\begin{array}{l} \overset{\infty }{\underset{n=0}{\sum }}\;\frac{{\left(\alpha_{1}\right)}_{nu_{1}}\cdots {\left(\alpha_{\ell }\right)}_{nu_{\ell }}}{{\left(\beta_{1}\right)}_{nv_{1}}\cdots {\left(\beta_{m}\right)}_{nv_{m}}}\;\Omega_{\lambda }(z,\omega ;s+n,a)\;\frac{t^{n}}{n!}\\ \;=\overset{\infty }{\underset{k=0}{\sum }}\;\Xi_{k}\;{\left(1-\frac{\omega }{k+a}\right)}^{-\lambda }\\ \;\;\cdot_{\ell }\Psi_{m}^{*}\left[\begin{array}{c} \left(\alpha_{1},u_{1}\right),\cdots \;,\left(\alpha_{\ell },u_{\ell }\right);\\ \left(\beta_{1},v_{1}\right),\cdots \;,\left(\beta_{m},v_{m}\right); \end{array}\frac{t}{k+a}\right]\;\frac{z^{k}}{{\left(k+a\right)}^{s}}\\ \left(\ell ,m\in N_{0};\;\alpha_{j}\in C,\;u_{j}\in R^{+}\;\;(j=1,\cdots \;,\ell );\right.\\ \left.\beta_{j}\in C\setminus Z_{0}^{-},\;v_{j}\in R^{+}\;\;(j=1,\cdots \;,m);\;\max\{|\omega |,|t|\}<1\right), \end{array}$$

where the sequence ${\left\{\Xi_{n}\right\}}_{n\in N_{0}}$ of the coefficients is given by the definition (2.13) and it is *tacitly* assumed that each member of the generating relation (3.30) exists. We do, however, choose to leave the details involved in all such derivations as exercises for the interested reader.

## *τ*-Generalizations of the Hurwitz-Lerch Zeta functions

In a recent paper, Saxena *et al.* (2011a) considered a so-called *τ*-generalization of the Hurwitz-Lerch Zeta function Φ(*z*, *s*, *a*) in (1.1) in the following form [Saxena *et al.* ((2011a), p. 311, Equation (2.1))]:

4.1 $$\Phi (\tau ;z,s,a):=\overset{\infty }{\underset{n=0}{\sum }}\frac{z^{n}}{{\left(\mathrm{\tau n}+a\right)}^{s}}\;(\tau \in R^{+}).$$

Subsequently, by similarly introducing a parameter *τ*>0 in the definition (2.5), Gupta and Kumari (2011) studied a *τ*-generalization of the extended Hurwitz-Lerch Zeta function $\Phi_{\mu }^{*}(z,s,a)$ in (2.5) as follows:

4.2 $$\Phi_{\mu }^{*}(\tau ;z,s,a):=\overset{\infty }{\underset{n=0}{\sum }}\;\frac{{\left(\mu \right)}_{n}}{n!}\;\frac{z^{n}}{{\left(\mathrm{\tau n}+a\right)}^{s}}\;(\tau \in R^{+}),$$

which, when compared with the definition (4.1), yields the relationship:

4.3 $$\Phi (\tau ;z,s,a)=\Phi_{1}^{*}(\tau ;z,s,a)\;(\tau \in R^{+}).$$

By looking closely at the definitions (4.1) and (4.2), in conjunction with the earlier definitions (1.1) and (2.5), respectively, we immediately get the following rather obvious connections:

4.4 $$\begin{array}{cc} \Phi (\tau ;z,s,a) & =\frac{1}{\tau^{s}}\;\Phi \left(z,s,\frac{a}{\tau }\right)\;\text{or}\;\\ \Phi \left(z,s,a\right) & =\tau^{s}\;\Phi (\tau ;z,s,\mathrm{a\tau})\;(\tau \in R^{+}) \end{array}$$

and

4.5 $$\begin{array}{cc} \Phi_{\mu }^{*}(\tau ;z,s,a) & =\frac{1}{\tau^{s}}\;\Phi_{\mu }^{*}\left(z,s,\frac{a}{\tau }\right)\;\text{or}\;\\ \Phi_{\mu }^{*}\left(z,s,a\right) & =\tau^{s}\;\Phi_{\mu }^{*}(\tau ;z,s,\mathrm{a\tau})\;(\tau \in R^{+}) \end{array}$$

Clearly, therefore, the definitions in (4.1) and (4.2) (*with*$\tau \in R^{+}$) are no more general than their corresponding well-known cases when *τ* = 1 given by the definitions in (1.1) and (2.5), respectively. Thus, by *trivially* appealing to the parametric changes exhibited by the connections in (4.4) and (4.5), all of the results involving the so-called *τ*-generalized functions Φ(*τ*;*z*, *s*, *a*) and $\Phi_{\mu }^{*}(\tau ;z,s,a)$ can be derived simply from the corresponding (usually known) results involving the familiar functions Φ(*z*, *s*, *a*) and $\Phi_{\mu }^{*}(z,s,a)$, respectively. Just for illustration of the triviality associated with such straightforward derivations, we recall the following sum-integral representation formula due to Lin and Srivastava ((2004), p. 729, Equation (20)) (see also Srivastava *et al.* ((2011), p. 494, Equation (2.6)) for the special case when *k* = 1):

4.6 $$\begin{array}{l} \Phi_{\mu ,\nu }^{\left(\rho ,\sigma \right)}(z,s,a)\\ =\frac{1}{\Gamma (s)}\;\overset{k-1}{\underset{j=0}{\sum }}\frac{{\left(\mu \right)}_{\mathrm{\rho j}}}{{\left(\nu \right)}_{\mathrm{\sigma j}}}\;z^{j}\\ \;\cdot \int_{0}^{\infty }t^{s-1}\;e^{-(a+j)t}\;\;{}_{2}\Psi_{1}\left[\begin{array}{c} \left(\mu +\mathrm{\rho j},\mathrm{\rho k}\right),(1,1);\\ \left(\nu +\mathrm{\sigma j},\mathrm{\sigma k}\right); \end{array}z^{k}\;e^{-\text{kt}}\right]dt\\ \left(k\in N;\;\min\{ℜ(a),ℜ(s)\}>0;\;\sigma >\rho >0\;\text{when}\;\right.\\ \left.z\in C;\;\sigma ≧\rho >0\;\text{when}\;|z{|}^{1/k}<\rho^{-\rho }\;\sigma^{\sigma }\right), \end{array}$$

it being tacitly assumed that each member of (4.6) exists. Indeed, in the special case when *ρ*=*σ*=*ν*=1, (4.6) yields the following sum-integral representation for the generalized Hurwitz-Lerch Zeta function $\Phi_{\mu }^{*}(z,s,a)$ involved in (2.5):

4.7 $$\begin{array}{l} \Phi_{\mu }^{*}(z,s,a)\\ =\frac{1}{\Gamma (s)}\;\overset{k-1}{\underset{j=0}{\sum }}\frac{{\left(\mu \right)}_{j}}{{\left(\nu \right)}_{j}}\;z^{j}\\ \;\cdot \int_{0}^{\infty }t^{s-1}\;e^{-(a+j)t}\;{}_{2}\Psi_{1}\left[\begin{array}{c} \left(\mu +j,k\right),(1,1);\\ \left(\nu +j,k\right); \end{array}z^{k}\;e^{-\text{kt}}\right]dt\\ \left(k\in N;\;\min\{ℜ(a),ℜ(s)\}>0;\;|z|<1\right) \end{array}$$

or, equivalently,

4.8 $$\begin{array}{l} \Phi_{\mu }^{*}(z,s,a)\\ =\frac{1}{\Gamma (s)}\;\overset{k-1}{\underset{j=0}{\sum }}\frac{{\left(\mu \right)}_{j}}{{\left(\nu \right)}_{j}}\;z^{j}\\ \;\cdot \int_{0}^{\infty }\;\;t^{s-1}\;e^{-(a+j)t}{}_{k+1}F_{k}\left[\;\begin{array}{c} \Delta^{*}\left(k;\mu +j\right),(1,1);\\ \Delta^{*}\left(k;\nu +j\right); \end{array}z^{k}\;e^{-\text{kt}}\;\right]dt\\ \left(k\in N;\;\min\{ℜ(a),ℜ(s)\}>0;\;|z|<1\right), \end{array}$$

where, for convenience, *Δ*^∗^(*n*;*λ*) abbreviates the array of *n* parameters

$$\frac{\lambda }{n},\frac{\lambda +1}{n},\cdots \;,\frac{\lambda +n-1}{n}\;(n\in N),$$

the array being empty when *n* = 0.

Now, in order to rewrite this last result (4.8) in terms of the *τ*-generalized Hurwitz-Lerch Zeta function $\Phi_{\mu }^{*}(\tau ;z,s,a)$ defined by (4.2), we simply make the following parameter and variable changes:

$$a\mapsto \frac{a}{\tau },\;\;t\mapsto \mathrm{\tau t}\;\text{and}\;dt\mapsto \tau dt\;(\tau \in R^{+})$$

and multiply the resulting equation by *τ*^−*s*^. By using the connection in (4.5), we thus find immediately that

4.9 $$\begin{array}{l} \Phi_{\mu }^{*}(\tau ;z,s,a)\\ =\frac{1}{\Gamma (s)}\;\overset{k-1}{\underset{j=0}{\sum }}\frac{{\left(\mu \right)}_{j}}{{\left(\nu \right)}_{j}}\;z^{j}\\ \;\cdot \int_{0}^{\infty }\;\;t^{s-1}\;e^{-(a+\mathrm{\tau j})t}{}_{k+1}F_{k}\;\left[\;\;\begin{array}{c} \Delta^{*}\left(k;\mu +j\right),(1,1);\\ \Delta^{*}\left(k;\nu +j\right); \end{array}z^{k}\;e^{-\mathrm{k\tau t}}\;\;\right]\;dt\\ \left(k\in N;\;\min\{ℜ(a),ℜ(s)\}>0;\;|z|<1\right). \end{array}$$

In its particular case when *k* = 1, this last formula (4.9) would simplify at once to the following form given by Saxena *et al.* ((2011a), p. 311, Equation (2.2)):

4.10 $$\begin{array}{l} \Phi_{\mu }^{*}(\tau ;z,s,a)=\frac{1}{\Gamma (s)}\;\int_{0}^{\infty }t^{s-1}\;e^{-\text{at}}\;{\left(1-ze^{-\mathrm{\tau t}}\right)}^{-\mu }\;dt\;\\ \left(ℜ(a)>0;\;ℜ(s)\}>0\;\text{when}\;|z|<1;\;ℜ(s)>1\right.\;\;\\ \left.\text{when}\;z=1\right),\; \end{array}$$

which obviously is equivalent to (and certainly not a generalization of) of the *τ* = 1 case derived earlier by Goyal and Laddha ((1997), p. 100, Equation (1.6)).

### Remark 9

The so-called *τ*-generalizations ${}_{2}R_{1}^{\tau }$ and ${}_{1}R_{1}^{\tau }$ of the Gauss hypergeometric function _2_*F*_1_ and Kummer’s confluent hypergeometric function _1_*F*_1_, respectively, which were used in the aforecited paper by Saxena *et al.* ((2011a), p. 315), are obviously very specialized cases of the well-known and extensively-investigated Fox-Wright function _*p*_*Ψ*_*q*_ defined by (2.15). In fact, it is easily seen from Definition 2 that [Saxena *et al.* ((2011a), pp. 315 and 317)] (see also (Al-Zamel 2001), (Ali *et al.*^2001^) and (Virchenko *et al.*^2001^))

4.11 $$\begin{array}{ll} {}_{2}R_{1}^{\tau }(a,b;c;z) & :=\frac{\Gamma (c)}{\Gamma (b)}\;\overset{\infty }{\underset{n=0}{\sum }}\frac{{\left(a\right)}_{n}\Gamma (b+\mathrm{\tau n})}{\Gamma (c+\mathrm{\tau n})}\;\frac{z^{n}}{n!}\\ =\frac{\Gamma (c)}{\Gamma (a)\Gamma (b)}\;{}_{2}\Psi_{1}\left[\begin{array}{c} \left(a,1\right),(b,\tau );\\ \left(c,\tau \right); \end{array}z\right]\\ ={}_{2}\Psi_{1}^{*}\left[\begin{array}{c} \left(a,1\right),(b,\tau );\\ \left(c,\tau \right); \end{array}z\right]\\ \left(|z|<1;\;\tau \in R^{+};\;c\notin Z_{0}^{-}\right) \end{array}$$

and

4.12 $$\begin{array}{ll} {}_{1}R_{1}^{\tau }(b;c;z) & :=\frac{\Gamma (c)}{\Gamma (b)}\;\overset{\infty }{\underset{n=0}{\sum }}\frac{\Gamma (b+\mathrm{\tau n})}{\Gamma (c+\mathrm{\tau n})}\;\frac{z^{n}}{n!}\\ =\frac{\Gamma (c)}{\Gamma (b)}\;{}_{1}\Psi_{1}\left[\begin{array}{c} \left(b,\tau \right);\\ \left(c,\tau \right); \end{array}z\right]\\ ={}_{1}\Psi_{1}^{*}\left[\begin{array}{c} \left(b,\tau \right);\\ \left(c,\tau \right); \end{array}z\right]\\ (|z|<\infty ;\;\tau \in R^{+};\;c\notin Z_{0}^{-}). \end{array}$$

Similar remarks and observations would apply equally strongly to the other *τ*-generalizations of well-known and extensively-investigated hypergeometric functions in one, two and more variables.

We conclude this section by presenting a generalization of the sum-integral representation formula (4.6) due to Lin and Srivastava ((2004), p. 729, Equation (20)).

### Theorem 6

The following sum-integral representation formula holds true:

4.13 $$\begin{array}{l} \Phi_{\lambda_{1},\cdots \;,\lambda_{p};\mu_{1},\cdots \;,\mu_{q}}^{\left(\rho_{1},\cdots \;,\rho_{p},\sigma_{1},\cdots \;,\sigma_{q}\right)}(z,s,a)=\frac{1}{\Gamma (s)}\;\overset{k-1}{\underset{j=0}{\sum }}\frac{\overset{p}{\underset{\ell =1}{\prod }}{\left(\lambda_{\ell }\right)}_{j\rho_{\ell }}}{\overset{q}{\underset{\ell =1}{\prod }}{\left(\mu_{\ell }\right)}_{j\sigma_{\ell }}}\;\frac{z^{j}}{j!}\;\int_{0}^{\infty }t^{s-1}\;e^{-(a+j)t}\;\\ \;\cdot_{p+1}\;\Psi_{q+1}^{*}\left[\begin{array}{c} \left(\lambda_{1}+j\rho_{1},k\rho_{1}\right),\cdots \;,\left(\lambda_{p}+j\rho_{p},k\rho_{p}\right),(1,1);\\ \left(\mu_{1}+j\sigma_{1},k\sigma_{1}\right),\cdots \;,\left(\mu_{q}+j\sigma_{q},k\sigma_{q}\right),(j+1,k); \end{array}\;z^{k}\;e^{-\text{kt}}\right]dt\;\\ \left(k\in N;\;\min\{ℜ(a),ℜ(s)\}>0\right),\; \end{array}$$

provided that each member of the assertion (4.13) exists.

### Proof

First of all, in light of the following elementary series identity:

$$\overset{\infty }{\underset{n=0}{\sum }}\;f\left(n\right)=\overset{k-1}{\underset{j=0}{\sum }}\overset{\infty }{\underset{n=0}{\sum }}\;f\left(\text{kn}+j\right)\;\left(k\in N\right),$$

we find from the definition (2.12) that

4.14 $$\begin{array}{c} \Phi_{\lambda_{1},\cdots \;,\lambda_{p};\mu_{1},\cdots \;,\mu_{q}}^{\left(\rho_{1},\cdots \;,\rho_{p},\sigma_{1},\cdots \;,\sigma_{q}\right)}(z,s,a)=k^{-s}\;\overset{k-1}{\underset{j=0}{\sum }}\frac{\overset{p}{\underset{\ell =1}{\prod }}{\left(\lambda_{\ell }\right)}_{j\rho_{\ell }}}{\overset{q}{\underset{\ell =1}{\prod }}{\left(\mu_{\ell }\right)}_{j\sigma_{\ell }}}\;\frac{z^{j}}{j!}\\ \;\;\cdot \Phi_{\lambda_{1}+j\rho_{1},\cdots \;,\lambda_{p}+j\rho_{p},1;\mu_{1}+j\sigma_{1},\cdots \;,\mu_{q}+j\sigma_{q},j+1}^{\left(k\rho_{1},\cdots \;,k\rho_{p},1,k\sigma_{1},\cdots \;,k\sigma_{q},k\right)}\left(z^{k},s,\frac{a+j}{k}\right)\\ \;(k\in N). \end{array}$$

The assertion (4.13) of Theorem 6 would now emerge readily upon first appealing to the aforementioned known result due to Srivastava *et al.* ((2011), p. 504, Equation (6.4)) (see also Remark 3 above) given by

4.15 $$\begin{array}{l} \Phi_{\lambda_{1},\cdots \;,\lambda_{p};\mu_{1},\cdots \;,\mu_{q}}^{\left(\rho_{1},\cdots \;,\rho_{p},\sigma_{1},\cdots \;,\sigma_{q}\right)}(z,s,a)\\ \;=\frac{1}{\Gamma (s)}\int_{0}^{\infty }\;\;t^{s-1}\;e^{-\text{at}}{}_{p}\Psi_{q}^{*}\left[\;\begin{array}{c} (\lambda_{1},\rho_{1}),\cdots \;,(\lambda_{p},\rho_{p});\\ (\mu_{1},\sigma_{1}),\cdots \;,(\mu_{q},\sigma_{q}); \end{array}\;ze^{-t}\;\right]\;dt\\ \left(\min\{ℜ(a),ℜ(s)\}>0\right) \end{array}$$

and then setting

t↦ktanddt↦kdt(k∈N).

□

Obviously, in its special case when

$$p=2\;(\lambda_{1}=\mu \;\text{and}\;\rho_{1}=\rho ;\;\;\lambda_{2}=1\;\text{and}\;\rho_{2}=1)$$

and

$$q=1\;(\mu_{1}=\nu \;\text{and}\;\sigma_{1}=\sigma ),$$

the general result (4.13) asserted by Theorem 6 would reduce immediately to the known sum-integral representation formula (4.6) due to Lin and Srivastava ((2004), p. 729, Equation (20)).
